# *In Vitro* Cytotoxicity and Setting Time Assessment of Calcium-Enriched Mixture Cement, Retro Mineral Trioxide Aggregate and Mineral Trioxide Aggregate

**DOI:** 10.22037/iej.v12i4.16275

**Published:** 2017

**Authors:** Tahereh Pornamazeh, Zahra Yadegari, Amir Ghasemi, Seyedeh Mahsa Sheykh-al-Eslamian, Shiva Shojaeian

**Affiliations:** a *Dental School, Shahid Beheshti University of Medical Sciences, Tehran, Iran; *; b *Dental Research Center, Research Institute of Dental Sciences, Dental School, Shahid Beheshti University of Medical Sciences, Tehran , Iran*

**Keywords:** Biocompatibility, Calcium-Enriched Mixture, Mineral Trioxide Aggregate, Retro MTA, Setting Time

## Abstract

**Introduction:**

The present study sought to evaluate and compare biocompatibility and setting time of Retro mineral trioxide aggregate (MTA), calcium-enriched mixture (CEM) and Angelus MTA.

**Methods and Materials:**

CEM cement, Angelus MTA and Retro MTA were assessed in set and fresh states. Extracts transformed to each cavity of three 24-well plates in which 1×10^4^ cell were seeded into each well 24 h earlier. All specimens were incubated in a humidified incubator with 5% CO2 at 37^°^C. Mosmann’s tetrazolium toxicity (MTT) assay was used to determine *in vitro* cytotoxicity on L929 mouse fibroblast cell line. Cell viability was determined at 1, 24, and 72 h after exposure. The initial setting time was measured by 113.4 g Gilmore needle testing. Then, final setting times were assessed by the 456.5 g Gilmore needle. Data comparisons were performed using the analysis of variance (ANOVA) and Tukey's post hoc test (*α*=0.05).

**Results:**

All groups in both forms indicated higher cell vitality compared to positive control group (*P*<0.001). After 24 h, the set Retro MTA showed better biocompatibility compared to set CEM and set Angelus MTA (*P*<0.001). Retro MTA showed significantly lower initial and final setting time compared to CEM and Angelus MTA (*P*<0.001).

**Conclusion:**

Our results indicated the good cell viability values of Retro MTA and relatively short period of setting time. It seems a promising alternative material in clinical situations where accelerated setting is required. However, more clinical and *in vivo *investigations are needed for a clear decision making.

## Introduction

Mineral trioxide aggregate (MTA) is broadly used in various endodontic interventions including direct pulp capping, root end filling, restoration of resorptive defects, apexification and apexogenesis [1]. This biomaterial has many advantages; such as favorable sealing capacity, marginal adaptation, biocompatibility, and antibacterial impact [2, 3]. It induces cementogenesis, dentinogenesis, and osteogenesis [4-6]. However, lengthy clinical setting time, difficult handling, and high price are its disadvantages [7-12]. Thus, evolving new materials is still a challenging and interesting topic of investigation in this regard. 

Developing a perfect root-end filling or pulp capping material requires biocompatibility, dimensional stability, moisture resistance, long term stability, radiopacity and ease of handling [8, 9, 13]. To overcome the shortcomings of MTA, a diversity of novel calcium silicate based biomaterials are presented; such as calcium-enriched mixture (CEM) cement, Bioaggregate, Biodentine, EndoSequence, and Retro MTA [14]. 

CEM cement as a novel dental material is invented in Iran [8, 9]. It consists of various materials such as CaO (51.75% wt), SO_3_ (9.53% wt), P_2_O_5 _(8.49% wt), and SiO_2_ (6.32% wt). CEM cement not only demonstrated similar biocompatibility, initial pH, dimensional changes and working time to MTA [15-17], but also indicated several more benefits like decreased setting time, ease of handling, intensified flow and shorter film thickness [8, 18]. After mixing with water based solution bioactive phosphate and calcium-enriched substances form that can promote sealing ability of the material [8]. Moreover, CEM induces cell differentiation for hard tissue formation [19]. Previous studies showed favorable biological responses of human gingival fibroblasts to CEM [20] and histological evaluations represented equal inflammatory reactions compared with MTA [21]. 

Another new material recently presented as a hydraulic bioceramic is Retro MTA (BioMTA, Seoul, Korea). It is a combination of hydrophilic materials in which are not originated from Portland cement. Retro MTA includes calcium carbonate, aluminum oxide, silicon dioxide and hydraulic calcium zirconia. It is recommended by the manufacturer for restoration of root resorption and perforations, pulp capping and retro filings. They declared Retro MTA as an aesthetic filling material due to lack of discoloration [22]. Furthermore, the manufacturer claimed the initial setting takes place only in 150 sec [14]. However, there are few studies in this regard [22-25]. These studies mainly focused on physicochemical properties of this material and up to our knowledge only two studies investigated its biocompatibility [22, 25]. The comparison between Retro MTA with MTA and CEM cement can be helpful to make evidence based decision upon the better substitutions. Hence, the current study sought to evaluate and compare biocompatibility and setting time of Retro MTA, CEM and Angelus MTA.

## Materials and Methods

***Specimen preparation***

CEM cement (BioniqueDent, Tehran, Iran), Angelus MTA (Angelus MTA, Londrina, Paraná, Brazil), and Retro MTA (BioMTA, Daejeon, Korea) were separately mixed based on the manufacturers' guidelines under sterile conditions. For each material two states (fresh or set) were investigated. The cytotoxicity of Retro MTA, Angelus MTA and CEM cement in fresh and set forms were investigated in this study. The sealers were prepared under aseptic conditions according to the manufacturers’ instructions. In set group each of materials was coated on the bottom each well of a 6-well cell culture plate (thickness>1 mm). All three sealers were maintained for 24 h at 37^°^C in a humid atmosphere under sterile conditions, so that the polymerization went to completion. In the fresh group, the sealers dispensed immediately after mixing into the wells of a six-well tissue culture plate. In order to perform the extraction of fresh and set sealers, according to ISO 10993-12:2012, 8 mL of DMEM media was added to related coated well and incubated at 37^°^C in a humid atmosphere of 5% CO_2_. After 24 h, the plates were removed from the incubator, and the elute was ﬁltered (ﬁlters: Pore size 0.22 µm, Schleicher and Schuell, Dassel, Germany) [26]. 

***Cell culture***

Mouse L929 ﬁbroblasts cell line (forth passage) were seeded into three 96-well tissue culture plates at concentration of 4000 cells per well. In completed media included Dulbecco’s Modiﬁed Eagle Medium (DMEM) (Life Technologies, Inc., Grand Island, NY, USA) supplemented with 10% fetal bovine serum, (FBS, Life Technologies, Inc., Grand Island, NY, USA), 100 U/mL Penicillin, and 100 µg/mL Streptomycin (Sigma-Aldrich, St. Louis, MO, USA), for 24 h at 37^°^C, 5% CO_2_ and 98% humidity. After 24 h, culture media was removed and 200 µL of extractions were replaced in wells related to six test groups. Distilled water and complete media were used as the positive and negative controls, respectively, instead of extraction. All of tests were repeated for six times. The plates were incubated at 37^°^C, 5% CO_2_ and 98% humidity for 1, 24, and 72 h, and then were evaluated by MTT assay. 

**Table 1:** Mean (SD) of percentage of cell viability within experimental groups at each time point

72 hours	24 hours	1 hour	State	Material
**81.81 (6.06)** **93.17 (7.06)**	85.85 (9.27) 100.80 (8.15)	96.47 (9.58) 95.25 (11.07)	**Fresh** **Set**	**Retro MTA**
**89.16 (1.02)** **90.80 (8.52)**	93.61 (5.35) 80.77 (12.59)	96.80 (7.26) 94.41 (4.18)	**Fresh** **Set**	**CEM**
**89.65 (4.94)** **83.39 (7.52)**	90.77 (5.38) 77.52 (6.55)	98.48 (3.05) 91.22 (3.93)	**Fresh** **Set**	**Angelus MTA**

**Table 2:** Mean (SD) of initial and final setting times (min) within experimental groups

Mean (SD) of final setting time	Mean (SD) of initial setting time	Material
**12.66 (3.05)**	3.24 (0.10)	**Retro MTA**
**78.66 (25.79)**	14.66 (2.08)	**CEM**
**83.66 (17.61)**	17.33 (0.57)	**Angelus MTA**

***MTT assay***

The MTT assay was carried out in a sterile area. The sealer extractions were removed from wells, and replaced with the sterile MTT solution (5mg/mL) diluted in cell culture media (1:10 ratio). Then, the plates were incubated for 2 h at 37^°^C at 95% humidity and 5% CO_2_. After that, the formazan crystals were dissolved in dimethyl sulfoxide. Then optical densities were measured at 570 nm, with 620 nm as a reference wavelength, using an ELISA plate reader (Anthos 2020, Austria).

***Setting time evaluation***

Based on the International Stands Organization (ISO) 6876 specification and the ASTM C266-03 standard test, all tests were performed under standardized controlled condition (at 95% humidity and 37^°^C temperature). All specimens were mixed under manufacturers' guideline and were placed into metallic form to form a cylinder like shape with 10 mm diameter and 2 mm thickness. All experimental and control groups were performed in triplicate. The 113.4 g Gilmore needle were utilised for testing the initial setting time. Then, final setting times were assessed by the 456.5 g Gilmore needle.

**Figure 1:**
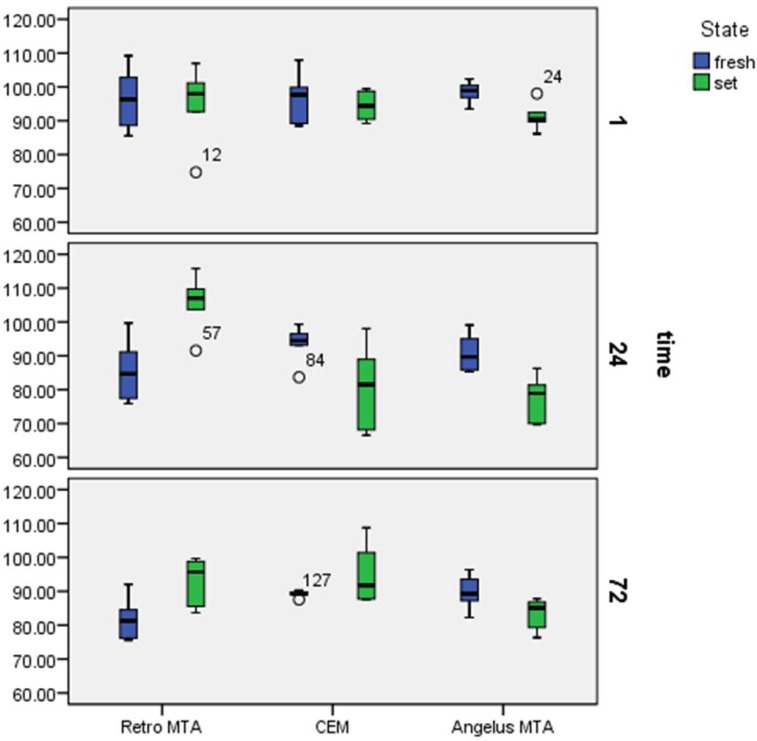
Boxplots of cell viability percentages in each experimental group at 1, 24, and 72 after exposure to the fresh or set groups’ extracts

**Figure 2:**
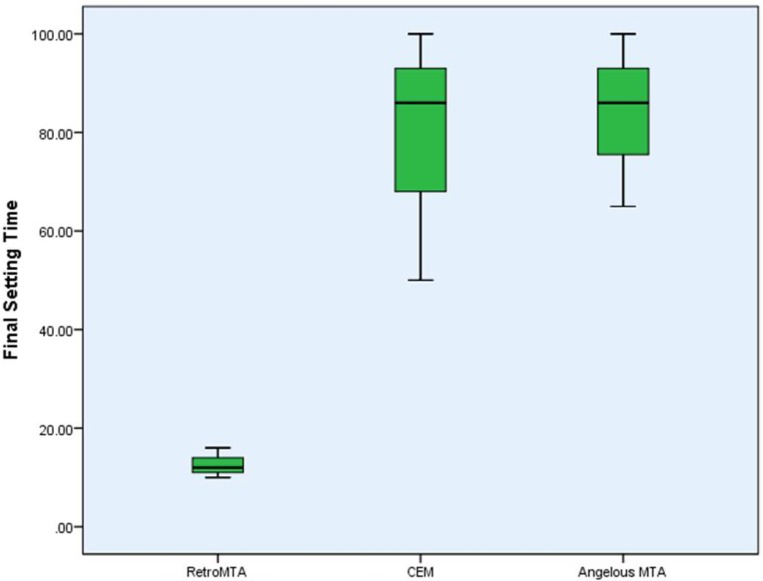
Boxplot of final setting time (minutes) for each experimental group

***Statistical analysis***

All data were analysed as mean (SD) of three independent exams and analysis performed using SPSS 20.0.1 software (IBM corp., Armonk, NY, USA). The ANOVA test followed by post-hoc Tukey’s tests were used for comparisons and the level of significance was set at 0.05. 

## Results

***Cell viability***

Table 1 and figure 1 demonstrates the percentages of cell viability assessed by MTT assay for each group. All groups in both forms indicated higher cell vitality compared to positive control group (*P*<0.001). The comparison between fresh form of materials did not show any significant differences between groups (*P*>0.001). However, there were some significant differences between set forms. After 24 h, the set Retro MTA showed better biocompatibility compared to set CEM and set Angelus MTA (*P*<0.001). Moreover, in all groups cell viability decreased over the time. 

***Setting time***

Initial and final setting times of each material are demonstrated in table 2. Retro MTA showed significantly lower initial and final setting time compare to CEM and Angelus MTA (*P*<0.001). The differences between CEM and Angelus MTA were not statistically significant at both initial and final measurements (*P*>0.001).

## Discussion

Nowadays, MTA is usually considered as the golden standard of endodontic material which its favorable biocompatibility is confirmed by many investigations [27-30]. So, newly presented materials often have been compared with it [13, 31]. In the present study, we assessed and compared the cytotoxicity of Retro MTA, CEM cement and Angelus MTA. In this study, the fresh form and set form of Retro MTA was compared to the others for the first time. The results represent no statistically differences between groups for the fresh type and significantly differences between Retro MTA and the others at 24 h and 72 h. However, none of them was not below the cut-off level established by ISO 10993-513 (70%) that showed all groups are biocompatible and improve cell viability compared to the positive control. 

In 2015, Souza *et al.* [22] compared the pH and cytotoxicity of Retro MTA (BioMTA, Seoul, Korea) with ProRoot MTA (Dentsply Tulsa Dental, Tulsa, OK, USA). In accordance to our results, they showed that both groups advanced the cell proliferation. However, lower cell vitality observed in Retro MTA group the difference was not significant. Although they utilized mitochondrial metabolism toxicity, lysossomal integrity and cell proliferation (DNA content) as biocompatibility tests, only the set form of materials was evaluated. 

The biocompatibility of CEM cement and MTA influenced by calcium ion release during their setting time and hydroxyapatite formation *via* calcium binding to phosphorus. These changes seem to initiate alterations in enzymatic activity of affected cells rather than permeability change [7, 27]. In addition, the fresh state applies clinically in root-end restorations. In agreement to our results Mozayeni *et al. *[32] indicated that no significant difference was seen between two form of MTA and CEM groups. However, Camilleri *et al.* [31]showed that fresh MTA has higher biocompatibility than its set state. On the other hand, Ghodussi *et al.* [33] found higher cytotoxicity in the fresh state compared with the set state of the same material during the follow-up intervals (24, 48 and 72 h).

Chung *et al. *[25] evaluated cell attachment and vitality in response to RetroMTA compared to ProRoot MTA and demonstrated similar effects between these groups. OrthoMTA as a very resembling material to RetroMTA based on their components, has been the subject of few studies [34-36]. In oppose to the previous results, Lee *et al.* [34] reported less biocompatibility of OrthoMTA compared to glass ionomer and ProRoot MTA. However, another article represented equal cell vitality and even cellular differentiation of OrthoMTA in comparison to Angelus MTA [35]. 

In some clinical conditions like repair of root perforations or retrograde filling, an accelerated setting time is required to evade dissolution of materials in blood and oral fluids. Despite many excellent biological and mechanical properties, MTA has long setting time that can be a disadvantage. Some alternative materials like CEM cement and RetroMTA can overcome this problem. Another objective of this study was to assess and compare the setting time of RetroMTA, CEM cement, and Angelus MTA. Based on our literature review, our study investigated setting time of RetroMTA for the first time. However, previous studies reported initial setting time of 12 min and final setting time of 140-170 min for Angelus MTA [20, 37-39]. Moreover, CEM cement has a shorter setting time for approximately 1 h [20] which is in agreement with our results . However, we did not find significant difference between CEM and MTA. Many factors can influence the setting time period. Addition of different components such as calcium sulfate can enhance the setting of Portland cement [40]. The shorter setting time of Retro MTA can be explained by its new composition. Although more investigations are needed to achieve evidence based decision toward material selection.

## Conclusion

Our results indicated favorable biocompatibility and quick setting time of Retro MTA compared to CEM cement and Angelus MTA. The good cell viability of Retro MTA and relatively short period of setting, present it as a promising alternative material in clinical situations in which required accelerated setting. However, more research is needed concentrating on the effect of this material on pulpal tissue and its mechanical properties.
